# Organizational adoption of AI-driven digital twins: evidence from the hospitality industry

**DOI:** 10.3389/frai.2026.1915911

**Published:** 2026-09-10

**Authors:** Tamara Gajić, Dragan Vukolić, Marko D. Petrović, Anastasiya A. Golubeva, Dunja Demirović Bajrami, Marija Bojić, Sergey V. Pashkov, Nesrin Atasoy, Tolga Kara, Emin Atasoy

**Affiliations:** 1 Geographical Institute «Jovan Cvijić » Serbian Academy of Sciences and Arts, Belgrade, Serbia; 2 Faculty of Organizational Studies–EDUKA, University Business Academy in Novi Sad, Belgrade, Serbia; 3 Faculty of Tourism and Hospitality Management, University of Business studies, Banja Luka, Bosnia and Herzegovina; 4 Department of Regional Economics and Geography, Faculty of Economics, RUDN University (Peoples’ Friendship University of Russia), Moscow, Russia; 5 Department of Economic Sociology, Faculty of Sociology, Saint Petersburg State University, Saint Petersburg, Russia; 6 Department of Geography, Tourism and Hotel Management, Faculty of Sciences, University of Novi Sad, Novi Sad, Serbia; 7 Faculty of Mathematics and Natural Sciences, M. Kozybayev North Kazakhstan University, Petropavlovsk, Kazakhstan; 8 Faculty of Humanities and Social Sciences, Sakarya University, Sakarya, Türkiye; 9 Ministry of National Education, Kocaeli, Türkiye; 10 Department of Social Sciences, Faculty of Education, Uludag University, Bursa, Türkiye

**Keywords:** central and Eastern Europe, digital readiness, digital transformation, hotel management, institutional support, technology acceptance

## Abstract

**Introduction:**

Artificial Intelligence (AI)-enabled Digital Twins are emerging as advanced cyber-physical technologies that support real-time decision-making, predictive analytics, and operational optimization. However, limited empirical evidence exists on the organizational factors influencing their adoption in hospitality, particularly across countries with different levels of digital maturity and institutional development. This study investigates hotel managers’ intentions to adopt AI-enabled Digital Twin technologies in Serbia and Hungary by extending the UTAUT2 framework with Digital Readiness and Institutional Support.

**Methods:**

A quantitative explanatory research design was employed. Data were collected from 492 hotel managers, including 234 respondents from Serbia and 258 from Hungary, who were involved in strategic decision-making concerning digital transformation and technological investment. The proposed model incorporated Performance Expectancy, Effort Expectancy, Social Influence, Facilitating Conditions, Digital Readiness, and Institutional Support as predictors of Behavioral Intention. The model was tested using Partial Least Squares Structural Equation Modeling (PLS-SEM) with SmartPLS 4, while Multi-Group Analysis (MGA) was employed to examine cross-country difference.

**Results:**

The measurement and structural models demonstrated satisfactory reliability, validity, and model fit. The model explained 48.2% of the variance in Behavioral Intention in Serbia and 53.7% in Hungary. Performance Expectancy, Effort Expectancy, Social Influence, Facilitating Conditions, Digital Readiness, and Institutional Support significantly influenced managers’ intentions to adopt AI-enabled Digital Twin technologies, although their relative importance differed between the two countries. Effort Expectancy and Digital Readiness were stronger drivers in Hungary, whereas Social Influence and Institutional Support exerted greater influence in Serbia, indicating that adoption mechanisms vary according to the surrounding digital and institutional environment.

**Discussion:**

The findings demonstrate that the organizational adoption of AI-enabled Digital Twins cannot be explained solely by conventional technology-acceptance factors but also depends on organizational digital capabilities and the institutional environment. Extending UTAUT2 with Digital Readiness and Institutional Support therefore provides greater explanatory value for understanding AI-enabled technology adoption. The Serbia–Hungary comparison further indicates that the relative importance of adoption determinants is context-dependent rather than universal, highlighting the importance of institutional maturity when explaining organizational AI adoption.

## 1 Introduction

Artificial Intelligence (AI) is transforming how organizations collect, process, and utilize data for strategic and operational decision-making. Advances in machine learning, predictive analytics, Internet of Things (IoT), and intelligent cyber-physical systems enable real-time optimization, predictive management, and operational intelligence across multiple industries (Mihai et al., 2022; Lucchi, 2025; Buhalis et al., 2024; Güney and Döker, 2025). Among these technologies, Digital Twins represent advanced AI-enabled cyber-physical systems that integrate real-time data, predictive models, and simulation capabilities to support intelligent decision-making (Yuce, 2023; Singh et al., 2023; Ghansah, 2025). By combining AI, IoT, cloud computing, sensor networks, and advanced analytics, Digital Twins enable organizations to monitor, predict, and optimize operational performance (Florido-Benítez, 2024; Lucchi, 2025).

The hospitality industry represents a promising context for AI-enabled Digital Twin implementation because hotels continuously generate large volumes of operational, environmental, and customer-related data. By integrating these heterogeneous data streams into intelligent virtual models, Digital Twins can optimize energy consumption, predictive maintenance, resource allocation, revenue management, sustainability, and personalized guest experiences (EI3, 2023; Schneider Electric, 2024; Singh et al., 2023; HotelTechnologyNews, 2024; Mirai, 2024; Prabawati et al., 2024). Consequently, Digital Twins are increasingly recognized as strategic AI-enabled systems rather than merely digital visualization tools. Despite their potential, organizational adoption of AI-enabled Digital Twin systems remains uneven, particularly across Central and Eastern Europe. While technologically advanced markets have begun implementing Digital Twin applications, adoption in hospitality remains limited in countries characterized by lower levels of digital maturity and institutional support (Haller, 2016; Mari et al., 2022; Lazić et al., 2023). Hungary benefits from stronger digital infrastructure, more developed innovation policies, and participation in European digitalization initiatives, whereas Serbia continues to face institutional and infrastructural barriers that may influence organizational technology adoption (Filipiak et al., 2023; Gyurkó et al., 2024; Durmishi, 2024). As an EU member state, Hungary has achieved higher levels of digital maturity and institutional readiness, while Serbia, as an EU candidate country, is still progressing in these areas. These differences are also reflected in the digitalization of the hospitality sector, making the two countries a suitable comparative context for examining how digital maturity and institutional environments shape organizational adoption of AI-enabled Digital Twin technologies. Accordingly, the present study is intentionally situated within the specific institutional and geographical context of Serbia and Hungary rather than aiming to develop universally applicable conclusions. These two neighboring Central and Eastern European countries provide a meaningful comparative setting because they represent different stages of digital maturity, institutional readiness, and hospitality digitalization. Consequently, the findings should be interpreted within this particular regional context, while future studies are encouraged to validate the proposed framework in other countries and institutional environments.

Although the European Union has increasingly promoted tourism digitalization through initiatives such as Smart Tourism Destinations, the hotel industries of Central and Eastern Europe have achieved uneven levels of digital maturity (Buonincontri and Micera, 2016; Niewiadomski, 2016; Baggio et al., 2020).

Although previous studies have examined the technological architecture, applications, and benefits of Digital Twins in tourism and hospitality (Fan et al., 2022a, 2022b; Singh et al., 2023; Buhalis et al., 2023; Deng et al., 2024; Ivanov et al., 2025; Ashton et al., 2025), empirical evidence explaining organizational adoption remains limited. Existing research primarily emphasizes technological capabilities rather than the organizational and managerial factors driving adoption decisions and rarely incorporates contextual determinants such as Digital Readiness and Institutional Support (Nikopoulou et al., 2023; Zaharia and Georgescu, 2025). Moreover, although UTAUT and UTAUT2 have been widely applied in hospitality research, their application to AI-enabled Digital Twin adoption and comparative examination across institutional contexts remain underexplored (Papathomas et al., 2025; Yadav et al., 2025). Accordingly, this study addresses these gaps by extending UTAUT2 with Digital Readiness and Institutional Support and empirically comparing organizational adoption in two countries characterized by different levels of digital maturity and institutional development.

These gaps are particularly important because Digital Twins differ substantially from many conventional digital technologies. Their implementation requires not only favorable perceptions of usefulness and ease of use but also advanced technological infrastructure, organizational capabilities, interoperability between multiple digital systems, and supportive institutional environments (Jones et al., 2020). Consequently, the adoption of AI-enabled Digital Twins should be viewed as an organizational innovation process rather than simply the introduction of another digital tool. Understanding these mechanisms is essential for explaining how AI technologies diffuse across organizations operating under different institutional conditions. Against this background, the present study develops and empirically tests an extended UTAUT2 framework for explaining organizational adoption of AI-enabled Digital Twin technologies in the hospitality sector. The proposed model incorporates two contextual constructs - Digital Readiness and Institutional Support - to capture organizational and environmental conditions that may influence managerial adoption intentions beyond traditional technology acceptance determinants. By comparing Serbia and Hungary, two neighboring European countries characterized by contrasting levels of digital maturity and institutional development, this study provides a unique opportunity to examine how contextual environments shape organizational AI adoption.

This study makes several important contributions to the existing literature. First, it extends the UTAUT2 framework to the context of AI-enabled Digital Twin technologies, an area that has received limited empirical attention. Second, it introduces Digital Readiness and Institutional Support as contextual determinants of organizational AI adoption, thereby expanding traditional technology acceptance theory toward organizational innovation research. Third, by comparing two institutional environments with varying stages of digital maturity, the study demonstrates that the relative importance of adoption determinants is context-dependent rather than universal. Accordingly, the findings should be interpreted as evidence derived from two specific Central and Eastern European institutional settings, providing a foundation for future comparative research in other geographical contexts. Finally, beyond the hospitality sector, the proposed framework offers a transferable perspective for understanding organizational adoption of AI-enabled cyber-physical systems in digitally transforming industries. Accordingly, the study addresses the following research questions:

*RQ1:* What factors most strongly influence hotel managers’ intentions to adopt AI-enabled Digital Twin technologies?

*RQ2:* What roles do Digital Readiness and Institutional Support play in shaping organizational adoption of AI-enabled Digital Twins?

*RQ3:* How do these influences differ between Serbia and Hungary, reflecting contrasting levels of digital maturity and institutional development?

The remainder of this paper is organized as follows. Section 2 presents the theoretical background and develops the conceptual framework and research hypotheses. Section 3 describes the research methodology, including the sampling procedure, measurement instrument, and data analysis approach. Section 4 presents the empirical results, followed by a discussion of the findings and their theoretical and managerial implications. Finally, Section 5 concludes the paper by summarizing the main contributions, acknowledging the study’s limitations, and suggesting directions for future research.

## 2 Theoretical background

### 2.1 AI-enabled digital twins in hospitality

Digital Twin (DT) technology has emerged as one of the key AI-enabled cyber-physical systems supporting real-time monitoring, predictive analytics, and intelligent decision-making across industries (Yuce, 2023; Singh et al., 2023; Mihai et al., 2022; Lucchi, 2025). Originally developed for manufacturing, Digital Twins are increasingly applied in service industries, including tourism and hospitality, due to advances in AI, IoT, cloud computing, and big data analytics (Buhalis et al., 2023; Florido-Benítez, 2024).

Within the hospitality sector, AI-enabled Digital Twins have gained increasing attention due to their potential to improve both operational performance and customer experiences. Hotels continuously generate large volumes of data related to occupancy levels, energy consumption, facility operations, guest preferences, maintenance requirements, and service delivery processes. By integrating these heterogeneous data streams into a unified digital environment, Digital Twins enable real-time monitoring, predictive maintenance, resource optimization, revenue management, and personalized service delivery (Singh et al., 2023; Prabawati et al., 2024). For example, Digital Twins can simulate alternative operational scenarios, identify inefficiencies before they occur, predict equipment failures, optimize energy usage, and support more sustainable resource management practices. Within the hospitality sector, AI-enabled Digital Twins enable real-time monitoring, predictive maintenance, resource optimization, revenue management, and personalized service delivery (Singh et al., 2023; Prabawati et al., 2024). They also support sustainable resource management and customer-oriented service innovation through AI-driven analytics and immersive digital environments (Deng et al., 2024; Koo et al., 2023; Buhalis et al., 2024; Meena et al., 2024; Zainal Abidin et al., 2025; Khan and Khan, 2025).

Although AI-enabled Digital Twins offer substantial opportunities for innovation and operational improvement, their adoption in the hospitality sector remains limited. Existing research has primarily focused on technological architectures, applications, and expected benefits, while comparatively less attention has been devoted to the organizational factors influencing adoption decisions (Nikopoulou et al., 2023; Zaharia and Georgescu, 2025). Successful implementation requires advanced technological infrastructure, organizational digital capabilities, interoperability, continuous data integration, and supportive institutional environments (Jones et al., 2020; Buhalis et al., 2024). Consequently, Digital Twin adoption should be viewed as a complex organizational innovation process rather than simply the introduction of another digital tool. Understanding these organizational determinants provides the foundation for applying the Unified Theory of Acceptance and Use of Technology (UTAUT2) to explain managerial intentions toward AI-enabled Digital Twin adoption.

### 2.2 UTAUT2 and organizational technology adoption

Understanding why organizations adopt emerging technologies has become a central issue in information systems, innovation management, and digital transformation research. The Unified Theory of Acceptance and Use of Technology (UTAUT), proposed by Venkatesh et al. (2003), is one of the most widely applied frameworks for explaining technology adoption. It integrates elements of the Technology Acceptance Model (TAM), Theory of Planned Behavior (TPB), Theory of Reasoned Action (TRA), Motivational Model, and Innovation Diffusion Theory (IDT), providing a comprehensive explanation of technology adoption behavior (Venkatesh et al., 2003). UTAUT2 subsequently extended the original model by incorporating additional consumer-oriented determinants while retaining its core constructs (Venkatesh et al., 2003). Performance Expectancy refers to the degree to which individuals believe that using a technology improves job performance (Venkatesh et al., 2003). It consistently represents one of the strongest predictors of technology adoption across organizational settings (Dwivedi et al., 2019; Foroudi et al., 2025a, b). In hospitality, managers are more likely to adopt technologies that enhance operational efficiency, service quality, cost reduction, and competitive advantage. Effort Expectancy reflects the perceived ease of learning and using a technology (Venkatesh et al., 2003). Technologies perceived as user-friendly generally face lower implementation resistance. Previous hospitality studies show that ease of use positively influences managerial attitudes toward innovation, particularly when advanced digital systems require organizational adaptation (Buhalis et al., 2023; Tul-Krzyszczuk, 2022). Although organizational technology adoption is frequently examined using frameworks such as the Technology Organization Environment (TOE) framework or Diffusion of Innovation (DOI), the present study adopts UTAUT2 because the focal unit of analysis is hotel managers’ behavioral intention to adopt AI-enabled Digital Twin technologies. Since adoption decisions are initiated and shaped by managerial perceptions, UTAUT2 provides an appropriate theoretical foundation for explaining these intentions. To better capture the organizational context, the model is extended by incorporating Digital Readiness and Institutional Support, thereby integrating organizational and environmental considerations emphasized in broader organizational adoption theories.

Social Influence refers to the perceived impact of colleagues, supervisors, professional communities, and other stakeholders on technology adoption decisions (Venkatesh et al., 2003). Within organizational settings, managerial decisions are shaped not only by perceptions of technology characteristics but also by social norms, industry trends, and external expectations. In the hospitality sector, professional associations, competitors, customers, and institutional actors can significantly influence managers’ attitudes toward digital transformation initiatives (Filimonau et al., 2024).

Facilitating Conditions refer to the availability of technological infrastructure, technical expertise, organizational support, financial resources, and training necessary for effective technology adoption (Venkatesh et al., 2003). Organizations with stronger technological capabilities and support systems are more likely to successfully implement complex innovations (Foroudi et al., 2025a). In hospitality, these conditions are particularly important because digital transformation requires substantial investments in technological infrastructure and organizational capabilities.

The UTAUT framework has been extensively applied in tourism and hospitality research to explain the adoption of digital technologies, including online booking systems, mobile applications, smart tourism technologies, artificial intelligence, service robots, virtual reality, and digital platforms (Buhalis et al., 2023; Ivanov et al., 2025; Papathomas et al., 2025; Flavián et al., 2019). These studies generally confirm its predictive validity across different technological and cultural contexts. However, the application of UTAUT to AI-enabled Digital Twin adoption remains limited. Unlike many digital technologies previously examined in hospitality, Digital Twins require not only favorable technology perceptions but also broader organizational capabilities, technological infrastructure, process integration, and strategic commitment (Jones et al., 2020; Singh et al., 2023). Accordingly, recent digital transformation research emphasizes that organizational capabilities and institutional environments are critical determinants of technology adoption (Verhoef et al., 2021; Vial, 2019). Therefore, this study extends the UTAUT2 framework by incorporating Digital Readiness and Institutional Support as contextual determinants of managers’ intentions to adopt AI-enabled Digital Twin technologies in the hospitality sector. These constructs are presented in the following section.

### 2.3 Digital readiness and institutional support as contextual determinants

While UTAUT2 provides a strong theoretical foundation for explaining technology adoption, recent studies suggest that organizational decisions regarding advanced digital technologies are also influenced by technological, organizational, and environmental factors (Vial, 2019; Verhoef et al., 2021). Accordingly, this study extends the UTAUT2 framework by incorporating Digital Readiness and Institutional Support as contextual determinants of managers’ adoption intentions.

Digital Readiness refers to the extent to which an organization possesses the technological infrastructure, digital competencies, and organizational capabilities required to successfully implement advanced digital technologies. Previous studies indicate that organizations with higher levels of digital maturity are more likely to adopt and benefit from emerging technologies (Jones et al., 2020; Kane et al., 2015; Verhoef et al., 2021; Buhalis et al., 2024). Therefore, Digital Readiness is expected to positively influence managers’ willingness to adopt AI-enabled Digital Twin technologies. Institutional Support refers to the extent to which regulatory frameworks, public policies, industry initiatives, financial incentives, and support programs facilitate technological innovation within organizations. Institutional theory argues that organizational decisions are embedded in broader socio-economic environments that may encourage or constrain innovation (Scott, 2014). This influence is particularly important in transition economies, where organizations often rely on public initiatives and institutional stability when evaluating investments in advanced technologies (Filimonau et al., 2024). Consequently, stronger Institutional Support is expected to increase managers’ intentions to adopt AI-enabled Digital Twin technologies. Together, Digital Readiness and Institutional Support complement the traditional UTAUT2 constructs by capturing organizational and environmental conditions that influence the adoption of complex AI-enabled technologies in the hospitality sector. Although Digital Readiness, Facilitating Conditions, and Institutional Support all support technology adoption, they represent conceptually distinct constructs. Facilitating Conditions capture managers’ perceptions of the availability of organizational resources and support required for technology use. Digital Readiness reflects the organization’s broader digital maturity and capability to implement and integrate advanced digital technologies. Institutional Support refers to external regulatory, policy, and industry conditions that facilitate organizational innovation. Accordingly, the three constructs represent operational, organizational, and environmental dimensions of technology adoption, respectively.

### 2.4 Conceptual framework and hypothesis development

Drawing upon the UTAUT2 framework and recent literature on digital transformation and organizational innovation, this study proposes a conceptual model explaining hotel managers’ intentions to adopt AI-enabled Digital Twin technologies. The model incorporates four core UTAUT2 determinants (Performance Expectancy, Effort Expectancy, Social Influence, and Facilitating Conditions) together with two contextual constructs, Digital Readiness and Institutional Support. The proposed framework assumes that managers’ adoption intentions are shaped not only by perceptions of the technology itself but also by the organizational and institutional environments within which adoption decisions occur. Performance Expectancy reflects managers’ beliefs that adopting AI-enabled Digital Twin technologies will improve organizational performance (Venkatesh et al., 2003). Previous empirical studies consistently identify Performance Expectancy as one of the strongest predictors of technology adoption intentions across different organizational contexts (Dwivedi et al., 2019). In tourism and hospitality, technologies perceived as improving operational efficiency, service quality, decision-making, and competitive advantage are more likely to be adopted by managers. Given the potential of Digital Twins to optimize hotel operations and enhance sustainability and customer experience, a positive relationship between Performance Expectancy and adoption intention is expected. Therefore:

*H1:* Performance Expectancy positively influences managers’ Behavioral Intention to adopt AI-enabled Digital Twin technologies.

Effort Expectancy refers to the perceived ease of understanding, implementing, and using a technology (Venkatesh et al., 2003). Previous studies show that technologies perceived as easy to use are associated with stronger adoption intentions, particularly in hospitality where managers evaluate implementation complexity before investing in new digital solutions (Buhalis et al., 2023; Tul-Krzyszczuk, 2022). Because AI-enabled Digital Twins require organizational adaptation and employee engagement, lower perceived implementation complexity is expected to increase managers’ willingness to adopt them. Therefore:

*H2:* Effort Expectancy positively influences managers’ Behavioral Intention to adopt AI-enabled Digital Twin technologies.

Social Influence reflects the extent to which managers perceive that important stakeholders encourage the adoption of advanced technologies (Venkatesh et al., 2003). Previous hospitality studies demonstrate that industry peers, customers, professional associations, and institutional actors significantly shape managers’ attitudes toward digital transformation initiatives (Filimonau et al., 2024). As Digital Twins become increasingly associated with smart hospitality strategies, external expectations and industry trends are expected to positively influence managers’ adoption intentions. Therefore:

*H3:* Social Influence positively influences managers’ Behavioral Intention to adopt AI-enabled Digital Twin technologies.

Facilitating Conditions refer to the availability of technological infrastructure, organizational resources, technical expertise, and support necessary for technology adoption (Venkatesh et al., 2003). Previous research indicates that organizations with stronger technological capabilities and support systems are more likely to implement complex digital innovations successfully (Foroudi et al., 2025b). Since AI-enabled Digital Twin technologies require substantial technological resources, stronger Facilitating Conditions are expected to positively influence managers’ adoption intentions. Accordingly:

*H4:* Facilitating Conditions positively influence managers’ Behavioral Intention to adopt AI-enabled Digital Twin technologies.

Digital Readiness reflects an organization’s capability to implement and integrate advanced digital technologies into its operations. Empirical studies consistently show that organizations characterized by higher levels of digital maturity are more likely to adopt innovative technologies successfully (Kane et al., 2015; Verhoef et al., 2021). Because AI-enabled Digital Twins require advanced digital capabilities, organizations with greater Digital Readiness are expected to demonstrate stronger adoption intentions. Therefore:

*H5:* Digital Readiness positively influences managers’ Behavioral Intention to adopt AI-enabled Digital Twin technologies.

Institutional Support reflects the extent to which regulatory frameworks, public policies, financial incentives, and industry initiatives facilitate technological innovation. Institutional theory suggests that supportive institutional environments reduce uncertainty and encourage organizational innovation (Scott, 2014). Previous studies also emphasize the importance of institutional support for digital transformation, particularly in transition economies (Filimonau et al., 2024). Consequently, stronger Institutional Support is expected to increase managers’ intentions to adopt AI-enabled Digital Twin technologies.

*H6:* Institutional Support positively influences managers’ Behavioral Intention to adopt AI-enabled Digital Twin technologies.

## 3 Methodology

### 3.1 Data collection and study design

This research utilizes a quantitative explanatory approach to explore the determinants of AI-enabled Digital Twin adoption in the hospitality industry. Drawing upon UTAUT2 as its theoretical foundation, the study extends the model by integrating Digital Readiness and Institutional Support to capture broader organizational and environmental influences. The theoretical foundation builds upon the work of Venkatesh et al. (2003) and its subsequent applications in tourism, hospitality, digital transformation, and technological innovation (Buhalis et al., 2023; Singh et al., 2023; Foroudi et al., 2025a, 2025b). By extending UTAUT2, the study aims to explain not only managers’ behavioral intentions toward adopting AI-enabled Digital Twin systems but also the broader organizational conditions that facilitate AI-driven digital transformation. Unlike conventional digital technologies, AI-enabled Digital Twin systems require the integration of technological infrastructure, organizational capabilities, real-time data processing, and supportive institutional environments. Their implementation therefore represents a multidimensional organizational innovation process rather than a simple technology acceptance decision. This assumption is consistent with recent studies emphasizing that Digital Twins operate as intelligent cyber-physical systems whose successful implementation depends on both technological and organizational readiness (Buhalis et al., 2024; Singh et al., 2023).

In the research, the questionnaire was created based on previously verified scales, whereby the constructs of the UTAUT2 model were adapted to the specifics of the industry’s digital twins. Terminology has been modified to suit hotel operations. In this way, the basic concepts of the UTAUT2 model are consistently translated into the language and situations relevant to hotel processes. Based on the previous studies (Venkatesh et al., 2003; Singh et al., 2023; Prabawati et al., 2024), the Facilitating Conditions (FC), Performance Expectancy (PE), Effort Expectancy (EE), Social Influence (SI), and Behavioral Intention (BI) constructs are adapted. In addition to these constructs, two external factors, digital readiness (DR) and institutional support (IS), were introduced into the model as necessary additions in the context of transition economies. Digital readiness included the manager’s assessment of the hotel’s technical equipment, the level of digital skills of employees and the organization’s ability to integrate DT systems, in accordance with contemporary works on the travel industry’s digital maturity (Buhalis et al., 2024; Lazić et al., 2023). Institutional support is defined through the perception of regulatory frameworks, industry guidelines, support programs, and broader systemic conditions that enable the introduction of advanced technologies, which is consistent with institutional gap theory and the TOE framework (Filimonau et al., 2024; Ahmad and Rasheed, 2025). These scales are further adapted to the hotel context so that they accurately reflect the sector’s dependence on available infrastructure, public policies and institutional security (Table 1). The instrument was pretested with 25 hotel managers in Belgrade and Novi Sad before the study, to determine the level of clarity, terminology and contextual appropriateness of the items. The validation of experts was done in two phases. To address the content validity, the questionnaire was first reviewed by three professors working in tourism and hospitality management and two IT professionals with experience in digital transformation projects to assess its content validity and relevance and the construct alignment with the UTAUT2 framework. Second, their feedback was included by making slight changes to the way items were worded (e.g., changing technical terms to hospitality-specific terms, making terms like a digital twin transparent when it comes to hotel operations). This mechanism helped to guarantee that the final instrument was theoretically and practically readable by the hotel managers, hence, enhancing its face and content validity. The findings showed the transparency of the items and the correctness of the answer time. Reliability was preliminarily supported through Cronbach’s alpha values above 0.80.

**Table 1:** Measurement items and operationalization of the proposed AI-enabled digital twin adoption framework.

Construct	Measurement items
Performance expectancy (PE)	PE1. AI-enabled digital twin technologies would improve our hotel’s overall performance.
	PE2. AI-enabled digital twin technologies would increase guest satisfaction.
	PE3. AI-enabled digital twin technologies would reduce our hotel’s operational costs.
	PE4. AI-enabled digital twin technologies would improve managerial decision-making.
	PE5. AI-enabled digital twin technologies would enhance our hotel’s competitiveness.
Effort expectancy (EE)	EE1. Learning to use AI-enabled digital twin technologies would be easy for our staff.
	EE2. AI-enabled digital twin technologies would be easy to learn and use.
	EE3. The software supporting AI-enabled digital twin technologies would be user-friendly.
	EE4. Implementing AI-enabled Digital Twin technologies would fit well with our existing work processes.
	EE5. Our employees would easily adapt to using AI-enabled Digital Twin technologies.
Social Influence (SI)	SI1. Our customers expect hotels to adopt advanced digital technologies.
	SI2. Competing hotels are increasingly investing in advanced digital technologies.
	SI3. The growing adoption of AI-enabled digital twin technologies in the hospitality industry encourages our hotel to consider their implementation.
	SI4. Professional hotel associations encourage the adoption of emerging digital technologies.
	SI5. Digital transformation initiatives promoted by the European Union encourage our hotel to adopt advanced technologies.
Facilitating Conditions (FC)	FC1. Our hotel has the necessary technical infrastructure to implement AI-enabled Digital Twin technologies.
	FC2. External IT specialists are available to support the implementation of AI-enabled Digital Twin technologies when needed.
	FC3. Financial resources or external support mechanisms are available to facilitate the adoption of AI-enabled Digital Twin technologies.
	FC4. Top management provides adequate organize

Source: Adapted from (Venkatesh et al., 2003).

Data were collected between January and June 2025 using a purposive and partially stratified sampling strategy targeting middle- and upper-level managers employed in three-, four-, and five-star hotels located in major urban tourism destinations in Serbia and Hungary. Respondents were selected because they participate directly in strategic decision-making regarding digital transformation, technological investments, and organizational innovation within their hotels. The availability of respondents in both countries was facilitated through professional networks and collaborations with alumni of higher education institutions employed in the hospitality sector, enabling access to managers representing different hotel categories and management levels. The selection of Serbia and Hungary provides a valuable comparative setting due to their contrasting levels of digital maturity and institutional development. Hungary, as a member state of the European Union, benefits from stronger digital infrastructure investments and strategic support for tourism digitalization (Filipiak et al., 2023; Gyurkó et al., 2024). In contrast, Serbia, although undergoing significant digital transformation, continues to face institutional and infrastructural challenges that may influence organizational adoption of advanced technologies (Durmishi, 2024). Previous studies have also demonstrated that digital maturity within Central and Eastern European tourism systems remains uneven, making these two neighboring countries an appropriate context for examining how institutional environments shape the adoption of AI-enabled Digital Twin technologies (Buonincontri and Micera, 2016; Niewiadomski, 2016; Baggio et al., 2020). Although purposive sampling was selected because the study required responses from hotel managers directly involved in technology adoption decisions, this approach may introduce selection bias and therefore limits the statistical generalizability of the findings. To improve sample representativeness, respondents were recruited from hotels of different categories, sizes, ownership structures, and geographical regions in both Serbia and Hungary. Consequently, although the sample is not statistically representative of the entire hotel population, it captures substantial organizational diversity relevant to the study objectives.

Approximately 698 managers were contacted across both countries, resulting in 492 valid questionnaires, including 234 respondents from Serbia and 258 respondents from Hungary, corresponding to an overall response rate of approximately 70.5%. Prior to statistical analysis, questionnaires containing more than 10% missing values or inconsistent response patterns were excluded from the dataset to ensure data quality and reliability. The hotel industry in Serbia comprises approximately 417 hotels, with the highest concentration located in Belgrade and a predominance of three-, four-, and five-star establishments. Hungary, by comparison, has approximately 3,034 commercial accommodation facilities, of which around 1,005 are hotels, reflecting a well-developed upper- and middle-market hospitality sector. These structural characteristics provide an appropriate empirical context for investigating managerial intentions regarding the adoption of AI-enabled Digital Twin technologies. The study was conducted in accordance with accepted ethical principles for social science research. Participation was voluntary, and respondents were informed of the study objectives as well as their right to withdraw at any stage without adverse consequences. To ensure confidentiality, no personally identifiable information was collected, and all analyses were performed using aggregated data.

### 3.2 Data analysis and measurement procedures

Partial Least Squares Structural Equation Modeling (PLS-SEM) using SmartPLS 4 was employed because it is particularly appropriate for studies with a predictive objective, complex research models, and data that may not satisfy the assumptions of covariance-based SEM (CB-SEM) (Hair et al., 2025; Kock, 2015). The proposed model extends the UTAUT2 framework by incorporating two additional contextual constructs, Digital Readiness and Institutional Support, resulting in multiple latent variables and structural relationships that increase model complexity. Moreover, the primary objective of this study is to explain and predict hotel managers’ behavioral intentions to adopt AI-enabled Digital Twin technologies rather than to confirm an established covariance structure. PLS-SEM is particularly suitable for maximizing the explained variance of endogenous constructs and for evaluating predictive relationships in theory extension studies. In addition, the data were collected using Likert-type scales and did not fully satisfy the assumption of multivariate normality, making PLS-SEM more appropriate than covariance-based SEM for the present analysis (Hair et al., 2025; Henseler et al., 2016; Kock, 2015). The Fornell-Larcker criterion and HTMT (< 0.85) were used to support discriminant validity, while factor loadings (≥ 0.70), AVE (≥ 0.50), and CR (≥ 0.70) were used to confirm convergent validity. Values as *R*^2^, effect sizes (*f*^2^), and predictive relevance (*Q*^2^) were implemented to evaluate the structural model. Significance of paths was tested with 5,000 bootstrap samples. To reduce the possibility of skewing the results due to common methodological variance, Harman’s univariate test was applied. As an additional check, the marker-variable approach was also applied, whereby no path in the model lost statistical significance after the inclusion of markers, which further confirms the absence of CMB effects. In addition to this, control variables (hotel category, facility size, and the manager’s years of experience) were included in the model as direct covariates toward the dependent variable (behavioral intention) in order to examine whether organizational and professional characteristics of managers alter the strength of the main structural paths in the model. None showed a statistically significant impact, nor did it change the significance of the main constructs, confirming the stability and robustness of the model. Alternative configurations of the model were also tested, in which the predictors were included and excluded individually, and the results remained consistent, indicating the structural stability of the estimation. An additional check of multicollinearity was conducted using Variance Inflation Factor (VIF). All outer VIF values ranged between 1.22 and 2.48, while inner VIF values were between 1.31 and 2.67. To additionally test for potential common method variance (CMV), a full collinearity test according to Kock (2015) was performed. All full collinearity VIF values were below 2.75, which is lower than the recommended cutoff of 3.3, indicating that CMV is not a threat to the validity of the results. Global model fit indices were also evaluated. The SRMR values indicate a good model fit (Serbia: SRMR = 0.061; Hungary: SRMR = 0.053), while the NFI values confirm satisfactory model alignment (Serbia: NFI = 0.924; Hungary: NFI = 0.935). In addition, RMS_theta values were acceptable (Serbia: 0.114; Hungary: 0.098), demonstrating adequate reliability. The diagnostics verify that the model is free of multicollinearity issues, and that global fit indices support the structural and measurement validity of the model.

Another specification was estimated to determine the strength and theoretical imperative of the extended model by dropping the contextual constructs Digital Readiness (DR) and Institutional Support (IS). The reduced UTAUT2-only model indicated significant reduction in the explained variance of Behavioral Intention (BI) where *R*^2^ decreased by 9.4% in Serbia and 11.2% in Hungary, which supports the idea that DR and IS have a significant positive predictive validity contribution. Their deletion also left behind systematic inflation of key path coefficients, especially effort expectancy and social influence meaning that the contextual constructs absorb the variance that otherwise would have been attributed to core UTAUT predictors (partial suppression effect). Even though none of the structural paths lost their significance, which proves that the underlying mechanisms underlying UTAUT are still present, the reduced model is theoretically incomplete and weaker empirically. Such findings demonstrate that the extended model fails without DR and IS, however, their inclusion gives it significantly worse results, which makes good sense (Table 2).

**Table 2:** Comparison of full and reduced PLS-SEM model specifications.

Metric	Full model (UTAUT2 + DR + IS)	Reduced model (UTAUT2 Only)	Change	Interpretation
*R*^2^ (BI)–Serbia	0.482	0.388	−0.094	Predictive power decreases without DR and IS
*R*^2^ (BI)–Hungary	0.537	0.425	−0.112	Reduced model substantially weaker
*β* shifts (EE → BI)	0.285	0.331	+0.046	Inflation due to removal of contextual constructs (suppression effect)
*β* shifts (SI → BI)	0.415	0.468	+0.053	Contextual constructs absorb shared variance
Model stability	All paths significant	All paths significant	Stable	Core UTAUT relations remain intact
Overall evaluation	Higher accuracy, theoretically complete	Lower accuracy, theoretically incomplete	—	Full model clearly superior

Source: Authors’ own elaboration.

Additionally, moderation analysis was done with hotel size, hotel category and managerial experience as the possible moderators of the relationships that predicted Behavioral Intention. All the interaction terms were not statistically significant (*p* > 0.05), which means that the strengths of the structural paths are similar in regard to various organizational and managerial characteristics. These findings indicate that technological and institutional factors are the main factors in the adoption of digital twins and not hotel profile or manager particularities. The non-significant moderating effects prove that the model does not change significantly depending on latent sample heterogeneity, which justifies the validity of the principal structural associations. Before conducting multiple group analysis (MGA), measurement invariance was tested using a three-step MICOM procedure. The first phase showed that configurational invariance was verified by confirming the same indicator specifications in both countries. In the second step, an analysis of compositional invariance was performed using permutation tests. In the third step, partial measurement invariance was confirmed, which enables a valid and statistically correct comparison of structural trajectories between Serbia and Hungary. This procedure ensured that any differences in MGA scores stemmed from substantive relationships among the constructs and not from measurement artifacts.

## 4 Empirical results

The empirical analysis was conducted in four stages. First, the demographic characteristics of the respondents were examined. Second, the reliability and validity of the measurement model were assessed. Third, the structural model and its predictive performance were evaluated using PLS-SEM. Finally, Multi-Group Analysis (MGA) was performed to compare the determinants of AI-enabled Digital Twin adoption between Serbia and Hungary.

Table 3 presents the demographic and professional characteristics of the respondents in Serbia and Hungary. The two samples are broadly comparable with respect to gender distribution, age structure, educational attainment, and hotel category, thereby providing a suitable basis for cross-country comparison. While managers in Hungary are more frequently employed in hotels located in the capital city, Serbian respondents are more evenly distributed across other major urban destinations.

**Table 3:** Demographic and professional characteristics of respondents.

Variable	Serbia (*n* = 234)	Hungary (*n* = 258)
Gender (Male)	58%	56%
Gender (Female)	42%	44%
Age (Mean)	41.9	40.6
Education (Bachelor’s)	45%	42%
Education (Master’s or higher)	55%	58%
Years in industry (Mean)	14.6	13.8
Hotel category (3-star)	33%	29%
Hotel category (4-star)	41%	45%
Hotel category (5-star)	26%	26%
Location (capital city)	54%	62%
Location (other cities)	46%	38%

Source: Authors’ own elaboration.

Following the assessment of descriptive statistics, the psychometric properties of the proposed AI-enabled Digital Twin adoption framework were evaluated through reliability and validity analyses before estimating the structural model. The assessment of measurement model reliability and convergent validity was first conducted for the Serbian sample. As shown in Table 4, the results indicate that all constructs met the established criteria for Cronbach’s alpha, Composite Reliability (CR), and Average Variance Extracted (AVE), thereby confirming adequate reliability and convergent validity. In addition, the standardized factor loadings are consistently high, confirming that the observed indicators adequately represent their respective latent constructs. Therefore, the Serbian measurement model demonstrates satisfactory psychometric properties and provides a reliable basis for structural model estimation.

**Table 4:** Psychometric properties of the measurement model (Serbia).

Construct	Item	*M*	*SD*	*λ*	*α*	CR	AVE
Performance expectancy (PE)	PE1	3.825	0.695	0.746	0.793	0.833	0.623
	PE2	3.793	0.732	0.747			
	PE3	3.832	0.715	0.771			
	PE4	3.876	0.691	0.815			
	PE5	3.788	0.711	0.796			
Effort expectancy (EE)	EE1	3.755	0.672	0.723	0.832	0.818	0.628
	EE2	3.729	0.689	0.918			
	EE3	3.873	0.702	0.903			
	EE4	3.789	0.677	0.872			
	EE5	3.803	0.708	0.731			
Social influence (SI)	SI1	3.799	0.661	0.728	0.812	0.832	0.638
	SI2	3.747	0.673	0.749			
	SI3	3.841	0.704	0.719			
	SI4	3.739	0.715	0.775			
	SI5	3.821	0.703	0.788			
Facilitating conditions (FC)	FC1	3.817	0.712	0.883	0.847	0.836	0.631
	FC2	3.712	0.721	0.835			
	FC3	3.816	0.719	0.776			
	FC4	3.781	0.683	0.723			
	FC5	3.766	0.694	0.772			
Digital readiness (DR)	DR1	3.62	0.71	0.743	0.812	0.858	0.603
	DR2	3.58	0.69	0.801			
	DR3	3.66	0.73	0.782			
	DR4	3.61	0.68	0.768			
Institutional support (IS)	IS1	3.42	0.74	0.721	0.794	0.846	0.578
	IS2	3.39	0.69	0.764			
	IS3	3.48	0.71	0.753			
	IS4	3.36	0.72	0.779			
Behavioral intention (BI)	BI1	3.796	0.731	0.715	0.802	0.851	0.644
	BI2	3.185	0.699	0.732			
	BI3	3.818	0.731	0.716			
	BI4	3.768	0.648	0.837			
	BI5	3.818	0.716	0.773			

Source: Authors’ own elaboration.

The same assessment was performed for the Hungarian sample. As presented in Table 5, all constructs again satisfy the recommended reliability and validity criteria, with Cronbach’s alpha, CR, and AVE values exceeding accepted thresholds. The standardized factor loadings also confirm the robustness of the measurement model, demonstrating that the latent constructs are consistently captured within the Hungarian sample. Collectively, these results indicate that the measurement model exhibits satisfactory reliability and allows meaningful comparisons across the two national contexts.

**Table 5:** Psychometric properties of the measurement model (Hungary).

Construct	Item	*M*	*sd*	*λ*	*α*	CR	AVE
Performance expectancy (PE)	PE1	4.174	0.607	0.871	0.897	0.884	0.692
	PE2	4.074	0.589	0.889			
	PE3	4.106	0.610	0.774			
	PE4	4.075	0.602	0.732			
	PE5	4.146	0.619	0.756			
Effort expectancy (EE)	EE1	4.121	0.584	0.783	0.823	0.874	0.638
	EE2	4.088	0.597	0.904			
	EE3	4.029	0.608	0.902			
	EE4	4.079	0.638	0.876			
	EE5	4.083	0.603	0.809			
Social influence (SI)	SI1	4.103	0.577	0.784	0.833	0.917	0.642
	SI2	4.223	0.623	0.836			
	SI3	4.019	0.615	0.837			
	SI4	4.115	0.616	0.817			
	SI5	4.098	0.582	0.728			
Facilitating conditions (FC)	FC1	4.105	0.609	0.895	0.842	0.894	0.663
	FC2	4.075	0.582	0.885			
	FC3	4.022	0.631	0.762			
	FC4	4.103	0.584	0.842			
	FC5	4.047	0.594	0.873			
Digital readiness (DR)	DR1	4.09	0.58	0.811	0.884	0.909	0.668
	DR2	4.11	0.61	0.854			
	DR3	4.07	0.57	0.782			
	DR4	4.15	0.59	0.829			
Institutional support (IS)	IS1	4.02	0.63	0.812	0.872	0.903	0.651
	IS2	4.11	0.60	0.857			
	IS3	4.07	0.59	0.812			
	IS4	4.05	0.62	0.781			
Behavioral intention (BI)	BI1	4.113	0.606	0.742	0.834	0.903	0.611
	BI2	4.139	0.605	0.819			
	BI3	4.038	0.607	0.831			
	BI4	4.034	0.586	0.712			
	BI5	4.126	0.605	0.873			

Source: Authors’ own elaboration.

After evaluating the measurement model, the overall quality and predictive performance of the proposed AI-enabled Digital Twin adoption framework were evaluated. As presented in Table 6, the model demonstrates satisfactory fit and reliability across both national samples. The Standardized Root Mean Square Residual (SRMR) values of 0.061 for Serbia and 0.053 for Hungary are below the recommended threshold of 0.08, indicating an acceptable model fit. Similarly, the Normed Fit Index (NFI) values of 0.924 and 0.935, respectively, further support the adequacy of the proposed model. The coefficient of determination (*R*^2^) shows that the framework explains 48.2% of the variance in Behavioral Intention (BI) among Serbian managers and 53.7% among Hungarian managers, indicating moderate to substantial explanatory power for organizational adoption of AI-enabled Digital Twin technologies. The slightly higher *R*^2^ observed for Hungary suggests that the proposed framework exhibits stronger predictive performance within a more digitally mature institutional environment. The construct reliability coefficients (ρA) are consistently high across all latent variables in both samples, confirming the internal consistency and stability of the measurement model. Moreover, the effect size statistics (*f*^2^) indicate that individual predictors contribute meaningfully to explaining Behavioral Intention, although their relative importance differs between the two countries. Overall, the results demonstrate that the proposed organizational AI adoption framework possesses satisfactory psychometric quality and predictive capability, thus supporting the reliability of the subsequent structural and multi-group analyses.

**Table 6:** Model fit and structural indices.

Country	SRMR	NFI	*R*^2^ (BI)	ρA_PE	ρA_EE	ρA_SI	ρA_FC	ρA_BI	*f*^2^_PE	*f*^2^_EE	*f*^2^_SI	*f*^2^_FC
Serbia	0.061	0.924	0.482	0.81	0.84	0.83	0.85	0.83	0.062	0.041	0.027	0.053
Hungary	0.053	0.935	0.537	0.88	0.90	0.91	0.89	0.90	0.083	0.058	0.036	0.064

Source: Authors’ own elaboration.

Discriminant validity of the proposed AI-enabled Digital Twin adoption framework was subsequently evaluated using the Fornell–Larcker criterion. As presented in Table 7, the square roots of the Average Variance Extracted (√AVE), displayed on the diagonal, are consistently higher than the corresponding inter-construct correlations for both the Serbian and Hungarian samples. This result indicates that each latent construct exhibits stronger associations with its own indicators than with other constructs, thereby supporting discriminant validity. The observed pattern indicates that the proposed constructs, including Performance Expectancy, Effort Expectancy, Social Influence, Facilitating Conditions, Digital Readiness, Institutional Support, and Behavioral Intention, represent conceptually distinct dimensions rather than overlapping phenomena. The similarity of the Fornell–Larcker matrices across the two national samples further suggests that the conceptual structure of the proposed organizational AI adoption framework is stable and comparable in both institutional contexts. Consequently, the measurement model satisfies the discriminant validity requirement and provides a reliable basis for estimating the structural relationships among the latent variables.

**Table 7:** Discriminant validity.

Construct	PE	EE	SI	FC	BI	DR	IS
PE	**0.79(S)/*0.83(H)***	*0.73*	*0.58*	*0.73*	*0.54*	*0.69*	*0.63*
EE	0.60	**0.79(S)/*0.80(H)***	*0.83*	*0.50*	*0.61*	*0.71*	*0.65*
SI	0.63	0.68	**0.80(S)/*0.80(H)***	*0.50*	*0.60*	*0.55*	*0.59*
FC	0.64	0.60	0.71	**0.79(S)/*0.81(H)***	*0.85*	*0.66*	*0.61*
BI	0.54	0.55	0.69	0.59	**0.77(S)/*0.78(H)***	*0.63*	*0.58*
DR	0.48	0.52	0.50	0.57	0.53	**0.78(S) / *0.82(H)***	*0.72*
IS	0.45	0.46	0.49	0.55	0.51	0.62	**0.76(S) / *0.80(H)***

Source: Authors’ own elaboration.

Note: S = Serbia; H = Hungary. Diagonal values in bold represent the square root of the Average Variance Extracted (√AVE). Values below the diagonal represent correlations for Serbia, while values above the diagonal represent correlations for Hungary.

Following confirmation of the measurement model, the structural relationships proposed in the conceptual framework were evaluated using PLS-SEM. Table 8 presents the estimated path coefficients (β), corresponding t-values, and significance levels for the Serbian and Hungarian samples. Overall, all hypothesized relationships are positive and statistically significant, providing empirical support for the extended UTAUT2 framework and confirming that managers’ intentions to adopt AI-enabled Digital Twin technologies are jointly influenced by technological, organizational, and institutional factors. In the Serbian sample, Social Influence emerged as the strongest predictor of Behavioral Intention (*β* = 0.415), followed by Facilitating Conditions (*β* = 0.300) and Effort Expectancy (*β* = 0.285). This finding suggests that hotel managers in Serbia are strongly influenced by external expectations, including industry peers, professional associations, and broader digital transformation trends. Such a pattern may reflect the relatively earlier stage of digital transformation in Serbia, where organizations often rely on external guidance and industry practices when evaluating emerging technologies. The significant effects of Facilitating Conditions and Effort Expectancy further indicate that the availability of organizational resources and the perceived ease of implementing Digital Twin technologies remain important considerations in managerial decision-making. Although Performance Expectancy exhibited the weakest effect among the core UTAUT2 constructs (*β* = 0.198), it remained statistically significant, indicating that managers also consider the expected organizational benefits of AI-enabled Digital Twins when forming adoption intentions. A different pattern was observed in the Hungarian sample. Effort Expectancy represented the strongest determinant of Behavioral Intention (*β* = 0.343), followed by Digital Readiness (*β* = 0.320), Social Influence (*β* = 0.271), Facilitating Conditions (*β* = 0.249), and Performance Expectancy (*β* = 0.244). The stronger influence of Effort Expectancy suggests that Hungarian managers place greater emphasis on the practical implementation and usability of AI-enabled Digital Twin technologies than on external social pressures. Furthermore, the relatively stronger effect of Digital Readiness indicates that organizational capabilities, digital competencies, and technological preparedness play a particularly important role in supporting the adoption of advanced AI technologies in a more digitally mature environment. These findings are consistent with the notion that organizations operating within more developed digital ecosystems increasingly depend on internal capabilities rather than solely on external influences when adopting technological innovations. Institutional Support exhibited a positive but comparatively weaker effect in both countries (Serbia: *β* = 0.180; Hungary: *β* = 0.150). This result suggests that although supportive regulatory frameworks, public policies, and government initiatives facilitate technology adoption, managerial decisions are driven more strongly by organizational capabilities and perceptions of the technology itself than by institutional incentives alone. Therefore, institutional support appears to function as an enabling rather than a primary determinant of AI-enabled Digital Twin adoption. Overall, the comparison between Serbia and Hungary demonstrates that the relative importance of adoption determinants differs across institutional contexts. While Serbian managers appear to rely more heavily on social influence and organizational support mechanisms, Hungarian managers place greater emphasis on organizational digital readiness and the ease of technology implementation. These findings indicate that organizational adoption of AI-enabled Digital Twin technologies is not governed by a universal set of determinants but is shaped by the broader digital maturity and institutional environment in which organizations operate. This provides empirical support for extending the UTAUT2 framework with Digital Readiness and Institutional Support and highlights the importance of considering contextual factors when explaining organizational AI adoption.

**Table 8:** Structural model path estimates.

Path	*β*	*t*-value	*p*-value
Serbia			
PE → BI	0.198	4.507	< 0.01
EE → BI	0.285	4.324	< 0.01
SI → BI	0.415	3.725	< 0.01
FC → BI	0.300	2.213	< 0.01
DR → BI	0.230	2.85	< 0.01
IS → BI	0.180	2.12	< 0.05
Hungary			
PE → BI	0.244	3.600	< 0.01
EE → BI	0.343	4.584	< 0.01
SI → BI	0.271	2.194	< 0.01
FC → BI	0.249	4.927	< 0.01
DR → BI	0.320	3.92	< 0.001
IS → BI	0.150	1.98	< 0.05

Source: Authors’ own elaboration.

Before conducting the Multi-Group Analysis (MGA), measurement invariance between the Serbian and Hungarian samples was assessed using the Measurement Invariance of Composite Models (MICOM) procedure. Establishing measurement invariance is an essential prerequisite for meaningful cross-group comparisons because it ensures that the latent constructs are conceptualized and measured equivalently across different populations. As presented in Table 9, the first step of the MICOM procedure, configural invariance, was satisfied for all constructs, indicating that both groups shared identical model specifications, indicators, and estimation procedures. The second step, compositional invariance, was also confirmed, as the correlation coefficients (c) exceeded the corresponding 5% permutation quantiles and none of the permutation tests indicated statistically significant deviations (all *p*-values > 0.05). Furthermore, the differences in construct means and variances between the two samples were not statistically significant, providing evidence of partial measurement invariance for all latent variables. According to the MICOM procedure, establishing partial measurement invariance is sufficient to permit valid comparisons of structural path coefficients using Multi-Group Analysis. Therefore, the Serbian and Hungarian samples can be considered directly comparable, allowing subsequent analyses to attribute observed differences to variations in structural relationships rather than to inconsistencies in measurement.

**Table 9:** MICOM measurement invariance assessment.

Construct	Configurational invariance	Compositional invariance (c)	5% quantile (cperm)	*p*-value (c)	Mean diff (p)	Variance diff (*p*)	Partial measurement invariance
PE	Yes	0.993	0.971	0.321	0.041	0.067	Yes
EE	Yes	0.991	0.968	0.287	0.058	0.074	Yes
SI	Yes	0.989	0.965	0.334	0.052	0.082	Yes
FC	Yes	0.992	0.969	0.305	0.047	0.071	Yes
BI	Yes	0.994	0.973	0.298	0.039	0.064	Yes
DR	Yes	0.987	0.961	0.356	0.049	0.078	Yes
IS	Yes	0.985	0.958	0.371	0.054	0.083	Yes

Source: Authors’ own elaboration.

Following confirmation of partial measurement invariance, a Henseler Multi-Group Analysis (MGA) was conducted to examine whether the structural relationships differed significantly between Serbia and Hungary. The results presented in Table 10 indicate that several determinants of Behavioral Intention vary significantly across the two countries, suggesting that organizational adoption of AI-enabled Digital Twin technologies is influenced by differences in digital maturity and institutional context. Specifically, Effort Expectancy exhibited a significantly stronger effect in Hungary (Δ*β* = 0.058; *p* < 0.05). This finding suggests that Hungarian hotel managers place greater importance on the ease of implementing and using AI-enabled Digital Twin technologies. In a more digitally mature environment, where the benefits of digital technologies may already be widely recognized, managers appear to focus primarily on implementation efficiency and operational usability when making adoption decisions. Similarly, Digital Readiness had a significantly stronger influence in Hungary (Δ*β* = 0.090; *p* < 0.05). This result indicates that organizational digital capabilities, technological infrastructure, and employee digital competencies play a particularly important role in facilitating the adoption of sophisticated AI-enabled technologies. As Digital Twins require substantial technological integration and organizational preparedness, this finding supports the argument that Digital Readiness becomes increasingly important as organizations progress in their digital transformation. Conversely, Social Influence exerted a significantly stronger effect in Serbia (Δ*β* = −0.144; *p* < 0.05). This suggests that Serbian managers rely more heavily on external influences, such as competitors, professional associations, industry practices, and stakeholder expectations, when evaluating the adoption of AI-enabled Digital Twin technologies. Such a pattern may reflect a context in which emerging technologies are still developing, making external validation and peer experience more influential in managerial decision-making. A similar pattern was observed for Facilitating Conditions, which demonstrated a significantly stronger effect in Serbia (Δ*β* = −0.051; *p* < 0.05). This finding indicates that the availability of technological infrastructure, technical expertise, and organizational support remains a more important prerequisite for technology adoption in Serbia than in Hungary. Managers operating in environments with lower levels of digital maturity may therefore place greater emphasis on the availability of implementation resources before committing to advanced AI technologies. In contrast, Performance Expectancy and Institutional Support did not differ significantly between the two countries (*p* > 0.05). This suggests that managers in both Serbia and Hungary similarly recognize the potential organizational benefits of AI-enabled Digital Twin technologies and perceive institutional support as a facilitating, rather than differentiating, factor in adoption decisions. The MGA results demonstrate that although the core determinants of AI-enabled Digital Twin adoption are significant in both countries, their relative importance varies according to the national digital and institutional environment. The findings therefore support the inclusion of Digital Readiness and Institutional Support as contextual extensions of the UTAUT2 framework and indicate that strategies for promoting AI-enabled Digital Twin adoption should be adapted to the specific characteristics of different institutional settings.

**Table 10:** Results of Henseler MGA analysis.

Path	*β* Serbia	*β* Hungary	Δ*β*	*p*-value	*f*^2^ Serbia	*f*^2^ Hungary	*Q*^2^ Serbia	*Q*^2^ Hungary
PE → BI	0.198	0.244	0.046	>0.05	0.10	0.10	0.29	0.33
EE → BI	0.285	0.343	0.058	<0.05	0.12	0.16	0.29	0.33
SI → BI	0.415	0.271	−0.144	<0.05	0.18	0.07	0.29	0.33
FC → BI	0.300	0.249	−0.051	<0.05	0.08	0.09	0.29	0.33
DR → BI	0.230	0.320	0.090	<0.05	0.07	0.13	0.29	0.33
IS → BI	0.180	0.150	−0.030	>0.05	0.04	0.03	0.29	0.33

Source: Authors’ own elaboration.

Conversely, Social Influence exerts a significantly stronger effect in Serbia (Δ*β* = −0.144; *p* < 0.05), while Facilitating Conditions also have a greater influence in the Serbian sample (Δ*β* = −0.051; *p* < 0.05). These findings indicate that managers operating in different institutional environments rely on different mechanisms when forming intentions to adopt advanced digital technologies. In contrast, the difference in Performance Expectancy between the two countries is not statistically significant (*p* > 0.05), suggesting that the perceived usefulness of AI-enabled Digital Twin technologies is relatively stable across contexts. Likewise, the difference in Institutional Support does not reach statistical significance (Δ*β* = −0.030; *p* > 0.05). The effect size statistics (*f*^2^) further illustrate these differences. In Serbia, Social Influence represents the strongest predictor (*f*^2^ = 0.18), whereas in Hungary the largest effect is observed for Effort Expectancy (*f*^2^ = 0.16). Moreover, the predictive relevance of the model is confirmed by positive *Q*^2^ values for both Serbia (*Q*^2^ = 0.29) and Hungary (*Q*^2^ = 0.33), demonstrating satisfactory out-of-sample predictive capability. Overall, the MGA results provide empirical evidence that the relative importance of technological, organizational, and social determinants of AI-enabled Digital Twin adoption varies across institutional settings, thereby justifying the use of a context-sensitive organizational AI adoption framework.

## 5 Discussion

The findings from the cross-country comparison indicate that the adoption of AI-enabled Digital Twin technologies in hospitality is a multifaceted process shaped by the combined effects of technological, organizational, and institutional determinants. Rather than being determined solely by the perceived characteristics of the technology itself, managers’ intentions to adopt Digital Twins depend on the broader digital ecosystem within which organizations operate. This finding supports the view that the implementation of AI-enabled systems should be understood as an organizational innovation process rather than merely a technology acceptance decision. The structural model confirms that Performance Expectancy, Effort Expectancy, Social Influence, Facilitating Conditions, Digital Readiness, and Institutional Support all significantly influence managers’ behavioral intentions. These findings are consistent with the assumptions of the UTAUT framework (Venkatesh et al., 2003) and with more recent studies emphasizing that the adoption of advanced digital technologies in hospitality is influenced simultaneously by technological perceptions and organizational context (Singh et al., 2023; Prabawati et al., 2024; Luther et al., 2023). However, the present results suggest that these determinants do not operate independently but interact with the institutional and digital environment in which organizations function.

Particularly important is the role of Digital Readiness, which extends the traditional technology acceptance perspective moving beyond personal perceptions of the technology’s utility and ease of implementation. The findings indicate that managers evaluate AI-enabled Digital Twin technologies not only according to their expected operational benefits but also according to the organization’s competence in integrating complex digital systems into existing business processes. This observation is consistent with studies on digital maturity and organizational transformation, which argue that successful implementation of advanced technologies depends on technological infrastructure, digital competencies, and organizational capabilities rather than on technological availability alone (Buhalis et al., 2024; Lazić et al., 2023; Durmishi, 2024). Similarly, the significance of Institutional Support confirms that the adoption of AI-enabled Digital Twin systems is embedded within a broader institutional environment. Regulatory frameworks, industry guidelines, financial incentives, and strategic digitalization policies reduce uncertainty associated with implementing emerging technologies and create conditions that facilitate organizational innovation. These findings are in accordance with studies emphasizing the importance of institutional ecosystems for digital transformation, particularly in transition economies where organizations frequently rely on external support mechanisms during technological change (Filimonau et al., 2024; Ahmad and Rasheed, 2025; Opoku et al., 2022).

The Multi-Group Analysis further demonstrates that the relative importance of adoption determinants differs between Serbia and Hungary, indicating that organizational AI adoption is strongly context-dependent. In Hungary, Effort Expectancy exerts a significantly stronger influence on Behavioral Intention, suggesting that in a more digitally mature environment managers focus primarily on the ease with which new technologies can be integrated into existing organizational processes. When basic technological infrastructure and institutional support are already established, implementation complexity becomes a major consideration in managerial decision-making. Similar observations have been reported in studies of digital transformation in hospitality, where technological compatibility and usability become increasingly important as digital maturity increases (Tul-Krzyszczuk, 2022; Rahmadian et al., 2023). In contrast, Serbian managers demonstrate significantly stronger dependence on Social Influence and Facilitating Conditions. This pattern suggests that in environments characterized by lower levels of digital maturity and less developed institutional ecosystems, managerial decisions are influenced more strongly by external expectations, peer practices, and the availability of organizational support mechanisms. Such findings are consistent with institutional theory and the concept of institutional voids, according to which organizations operating within less developed institutional environments compensate for uncertainty by relying more heavily on social norms and external legitimacy. Rather than evaluating technological characteristics alone, managers appear to consider whether sufficient organizational and environmental support exists for successful implementation.

An interesting finding is that Performance Expectancy does not differ significantly between the two countries. Regardless of differences in digital maturity, managers in both Serbia and Hungary similarly recognize the capacity of AI-enabled Digital Twin technologies to improve operational performance, reducing costs, optimizing resource allocation, and enhancing service personalization. This suggests that the perceived strategic value of Digital Twins is relatively universal, while the mechanisms through which organizations decide to implement such technologies vary across institutional contexts. Previous studies have likewise emphasized that expectations regarding long-term organizational benefits constitute one of the most stable drivers of advanced technology adoption in hospitality (Buhalis et al., 2024; Singh et al., 2023; Ghansah, 2025; Shan et al., 2025; Singh et al., 2025). Overall, the findings indicate that organizational adoption of AI-enabled Digital Twin technologies cannot be adequately explained by technological characteristics alone. Instead, adoption emerges from the interaction between expected technological benefits, organizational digital capabilities, social and institutional influences, and the broader digital maturity of the surrounding environment. Consequently, the results support a context-sensitive understanding of AI adoption, in which technological, organizational, and institutional dimensions jointly shape managerial intentions toward implementing advanced cyber-physical systems.

## 6 Conclusion

This research explored the organizational determinants of AI-enabled Digital Twin technology adoption within the hospitality sector by comparing hotel managers from Serbia and Hungary through an extended UTAUT2 framework incorporating Digital Readiness and Institutional Support. The findings demonstrate that managers’ intentions to adopt Digital Twin technologies are governed by a set of technological, organizational, and institutional factors rather than by technological perceptions alone. While the proposed model explains approximately half of the variance in Behavioral Intention in both countries, the relative importance of individual determinants differs according to the surrounding digital and institutional environment. The comparative analysis reveals that Effort Expectancy represents the strongest determinant of adoption in Hungary, suggesting that managers operating within a digitally mature ecosystem primarily evaluate the ease with which AI-enabled Digital Twin systems can be integrated into existing organizational processes. Conversely, Serbian managers rely more strongly on Social Influence and Facilitating Conditions, indicating that external support mechanisms and perceived organizational capabilities play a greater role in environments characterized by lower levels of digital maturity. At the same time, the relatively stable influence of Performance Expectancy across both countries suggests that the perceived strategic value of Digital Twin technologies is recognized regardless of institutional context.

At the theoretical level, the present research advances understanding of literature on organizational adoption of Artificial Intelligence by demonstrating that the implementation of AI-enabled Digital Twin technologies cannot be sufficiently explained by classical technology acceptance mechanisms alone. Although the UTAUT2 framework provides a robust basis for understanding behavioral intentions, the present findings show that the adoption of advanced AI systems is simultaneously conditioned by organizational capabilities and institutional ecosystems. By incorporating Digital Readiness and Institutional Support into the model, the study extends traditional technology acceptance theory beyond individual cognitive evaluations toward a broader organizational perspective on AI adoption. Furthermore, the findings suggest that the determinants of AI-enabled Digital Twin adoption are context-dependent rather than universal. While the perceived usefulness of the technology remains relatively stable across institutional environments, the varying influence of effort expectancy, social influence, facilitating conditions, and organizational readiness varies according to the level of digital maturity. This indicates that institutional environments function as enabling or constraining mechanisms that shape how managers evaluate and adopt AI technologies. Consequently, the study supports the growing argument that AI adoption should be conceptualized as an interaction between technological characteristics, organizational capabilities, and institutional conditions rather than as a purely individual decision-making process. The research also contributes to the literature on Digital Twins by positioning them not merely as technological tools for operational optimization, but as AI-enabled cyber-physical systems whose successful implementation depends on organizational transformation. Existing studies have primarily emphasized their technical architecture and potential applications, whereas empirical evidence explaining organizational adoption has remained limited. By empirically validating an extended UTAUT2 framework across two different institutional contexts, this study bridges the gap between engineering-oriented Digital Twin research and organizational AI adoption theory. Finally, the comparative design contributes to the broader debate on the generalizability of technology acceptance models. The significant differences identified between Serbia and Hungary demonstrate that behavioral drivers of AI adoption are moderated by national digital ecosystems and institutional maturity. These findings challenge the assumption that technology acceptance models operate uniformly across contexts and instead support the development of context-sensitive frameworks for organizational AI adoption. Therefore, the proposed model provides a transferable conceptual foundation for investigating the adoption of AI-enabled cyber-physical systems not only in hospitality but also in other digitally transforming service industries.

The study generates a number of practical implications for key stakeholders, including policymakers, hotel managers, technology providers, and destination management organizations, seeking to promote the effective adoption of AI-enabled Digital Twin technologies. The results indicate that successful implementation should not be viewed merely as the acquisition of a new technological solution but as a comprehensive organizational transformation process requiring the simultaneous development of technological infrastructure, digital competencies, and supportive institutional ecosystems. For hotel managers, the findings emphasize that investments in AI-enabled Digital Twin systems should be accompanied by employee training, organizational learning, and the integration of these technologies into existing operational and strategic decision-making processes. Simply introducing advanced technologies without ensuring organizational readiness is unlikely to generate their full potential benefits. Technology providers, in turn, should focus on developing user-friendly, interoperable, and easily integrated Digital Twin platforms that reduce implementation complexity and increase managerial confidence in adoption. The results are equally relevant for policymakers and public institutions responsible for digital transformation strategies. In digitally mature environments, policy measures should prioritize innovation support, interoperability standards, and the diffusion of best practices that facilitate the integration of AI technologies into hospitality operations. In contrast, countries characterized by lower levels of digital maturity require broader interventions aimed at strengthening digital infrastructure, financial support mechanisms, professional education, regulatory frameworks, and collaboration between public institutions, industry associations, and technology developers. Such coordinated actions can reduce uncertainty associated with AI implementation and accelerate the diffusion of Digital Twin technologies throughout the hospitality sector. More broadly, the findings suggest that the successful adoption of AI-enabled Digital Twins depends on the development of an enabling digital ecosystem rather than on technological availability alone. Consequently, strategies for AI implementation should combine investments in technology with organizational capacity building and institutional support in order to achieve sustainable digital transformation within the hospitality industry.

## 7 Limitations and future research

The findings of this study should be interpreted in light of several limitations. First, the study is based on a cross-sectional research design, preventing conclusions regarding causal relationships or changes in managerial perceptions over time. Second, the analysis relies on self-reported managerial assessments, which may be influenced by common method bias or individual optimism toward digital transformation. Third, although Serbia and Hungary provide a valuable comparison of two institutional contexts, the applicability of the findings to other geographical, institutional, and cultural settings should be approached with caution. Differences in digital maturity, regulatory frameworks, and technological infrastructure may influence the relative importance of the proposed determinants in other hospitality markets.

Future research should therefore employ longitudinal designs to examine how organizational AI adoption evolves as digital ecosystems mature and AI-enabled Digital Twin technologies become more widespread. Expanding comparative analyses to additional developed and emerging economies would further clarify the role of institutional context in shaping adoption mechanisms. Furthermore, qualitative approaches, including in-depth interviews, multiple case studies, and mixed-method research designs, could provide richer insights into organizational implementation processes, managerial decision-making, and barriers to AI-enabled Digital Twin adoption.

Although the extended UTAUT2 framework demonstrated satisfactory explanatory power, future studies could broaden the conceptual model by incorporating additional organizational, technological, and environmental determinants, such as organizational culture, innovation capability, data governance, cybersecurity readiness, leadership support, inter-organizational collaboration, and digital resilience. Future research may also refine and expand the measurement indicators to capture the multidimensional nature of organizational AI adoption more comprehensively. Moreover, investigating the interaction between AI-enabled Digital Twins and complementary technologies, including predictive analytics, generative AI, blockchain, Internet of Things, and intelligent service automation, would contribute to a more holistic understanding of integrated digital ecosystems within the hospitality industry.

## Data Availability

The raw data supporting the conclusions of this article will be made available by the authors, without undue reservation.
